# New Synonym and New Species Record of *Filchneria* (Plecoptera: Perlodidae) from China with a Morphological, Phylogenetic and Biogeographic Study on This Genus

**DOI:** 10.3390/insects13111044

**Published:** 2022-11-11

**Authors:** Qing-Bo Huo, Bin-Qing Zhu, Abdur Rehman, Dávid Murányi, Yu-Zhou Du, Jun Wu

**Affiliations:** 1 School of Horticulture and Plant Protection & Institute of Applied Entomology, Yangzhou University, Yangzhou 225009, China; 2 Department of Zoology, Eszterházy Károly Catholic University, Leányka u. 6, H-3300 Eger, Hungary; 3 Nanjing Institute of Environmental Sciences, Ministry of Ecology and Environment, Nanjing 210042, China; 4 Joint International Research Laboratory of Agriculture and Agri-Product Safety, the Ministry of Education, Yangzhou University, Yangzhou 225009, China

**Keywords:** Perlodidae, Plecoptera, *F. mongolica*, new record, China

## Abstract

**Simple Summary:**

When the perlodid genus *Filchneria* Klapálek, 1908 was proposed, *F. mongolica* (Klapálek, 1901) was designated as the type species, with a female holotype from Mongolia and another collection of male and female from the Qinling Mountains of central China. These specimens from different areas have been mistaken over the past one century. We verified this research history of the two amazing species by updating their morphological descriptions and distribution records. In addition, the genus *Sinoperlodes* Chen, 2020 recently proposed is proved to be a synonym of *Filchneria*, with the morphological, biological, phylogenetic and biogeographical issues discussed.

**Abstract:**

The type species of *Filchneria* Klapálek, 1908, *F. mongolica* (Klapálek, 1901), is based on a single female collected from Mongolia, but it was considered the same as another species, *F. songi* from Qinling, China, when the genus *Filchneria* was proposed. This study narrates the story of these two species, which have been confused for a century. Until now, the distribution of *F. mongolica* has been confirmed only in Mongolia and Russia, and we recently recorded it for the first time in Inner Mongolia as a new species record in China. Additionally, the genus *Sinoperlodes* is a junior synonym of *Filchneria*, as demonstrated by both the morphological and molecular analysis. Phylogenetic analysis based on the subfamily Perlodinae is provided, along with morphological and biogeographic comparisons of *Filchneria* and its relatives.

## 1. Introduction

*Filchneria* Klapálek, 1908 is known with 15 species distributed in the eastern part of the Palaearctic region, mainly in east and central Asia and the Caucasus mountains, and nine species has been recorded in China [1,2,3,4,5,6,7,8,9]. *Filchneria mongolica* (Klapálek, 1901), the type species of this genus, is also the first stonefly recorded in China.

**Figure 1:**
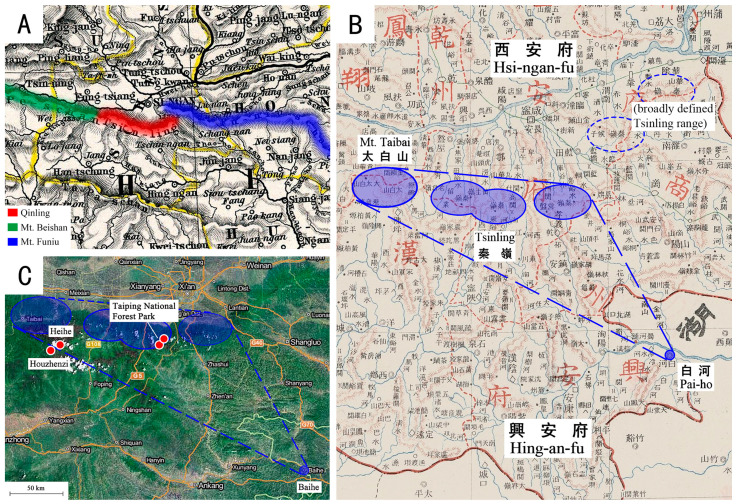
Qinling/Tsinling area with adjacent western and eastern mountains: (**A**) map modified at Stieler’s Hand-atlas, “Chinesisches Reich”, 1891, provided by Wikipedia; (**B**) the possible range from the Qinling to Baihe/Pai-ho is marked by blue dotted lines, Map of Shaanxi is modified by The Commercial Press [12]; (**C**) collecting localities of *F. songi*, map modified by (www.tianditu.gov.cn, accessed on 12 March 2020).

However, although the specific name of *F. mongolica* refers to “Mongolia”, this genus was proposed by Klapálek [2], according to a new collection from Tsinling (spelled in Wade-Giles system, which refers to the Qinling mountains), Shaanxi Province of China (Figure 1A). Klapálek [3] pointed out that the type materials of *F. mongolica* (one male, two females) were collected from “Tsinling am Weg Hsin-nganfu-Peiho, Coll. Filchner”, with another three females from Mongolia. Wu [4] also reported Klapálek’s record as “Types: One male and two females from Tsinling on the way to Hsin-Ngan-Fu … Paratypes: From N. Mongolia, deposited by Leder, in K. K. Hofmuseum. Three females from Issyk-kul, deposited by Tancre in Mus. Hamburg”. In fact, only the single female from N. Mongolia can be considered as the holotype, while the other specimens are not types. Hsin-ngan-fu [4] was mistaken by Du [10] for Hsi-ngan-fu (西安府, current Xi’an city), which is called “Kaiserstadt” by Erich von Salzmann [11]. Until recently, after the original record of Klapálek [2] had been checked, we found that the type locality of *F. mongolica*, “Ts’in-ling am Weg Hing-an-fu—Pai-ho” is actually determined as “the way from Qinling to Xinganfu (興安府), Baihe County (白河縣)” (Figure 1B). In 2019, we surveyed the stoneflies fauna in the Qinling Mountains from Mt. Taibai to Taiping Forest Park from March to April, and *Filchneria songi* Chen, 2019 was the only *Filchneria* species found there, with its morphology matching very well the description of *F. mongolica* (male and female from Qinling) by Klapálek [2].

**Figure 2:**
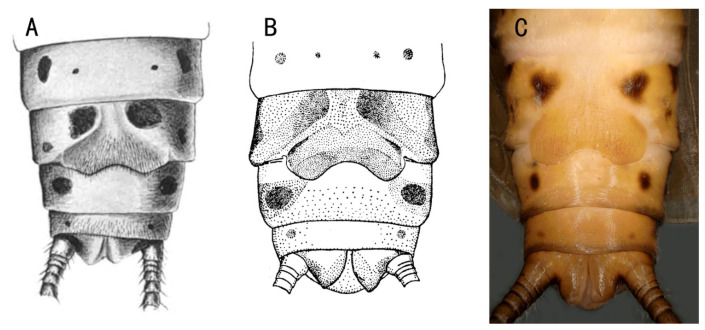
Terminalia of females, ventral: (**A**) *Filchneria mongolica*, in Klapálek [2]; (**B**) *Filchneria mongolica*, in Teslenko and Zhiltzova [6]; (**C**) *Filchneria songi*.

However, Chen [8] did not mention that Taiping Forest Park is very close to the locality recorded by Klapálek [2] (Figure 1C), and *Filchneria* was actually considered and proposed as a new genus after using the male and female adults from Qinling. We believe that Klapálek probably misidentified a similar female of a Mongolian congener for the same species as the specimen from Qinling due to their abdominal patterns and shapes of female subgenital plates (Figure 2), which are the most similar in this genus [6].

It means that the original materials of *F. mongolica* include at least two species separately from different areas, and *F. songi* from Qinling is the real type species that Klapálek [2] wanted to designate. It is incorrect to subjectively classify these species into the same taxon, which later led *F. mongolica* to be considered a widespread species from the Qinling Mountains to Siberia for more than one hundred years.

Until now, *F. mongolica* were only known from northeastern Mongolia and the Russian Far East [6,7,13]. There is no scientific evidence (specimen, photo, or believable collecting record) for the existence of this species in the Qinling Mountains and other Chinese areas. In this study, we recently discovered this species for the first time in Arxan City, Inner Mongolia Autonomous Region, and recorded it as a new distribution in China. Supplementary descriptions and color illustrations for *F. mongolica* and *F. songi* are provided in this paper.

After clarifying the above two *Filchneria* species, we considered another monotypic genus, *Sinoperlodes* Chen, 2020, to be a synonym of *Filchneria*. The type species of *Sinoperlodes*, *Sinoperlodes zhouchangfai* Chen, 2020, was recently reported from Zhejiang Province, China [14], and was also newly found in Anhui, Fujian, and Guizhou provinces. The morphology of its adult terminalia, larvae, and egg shows no differences from the typical diagnosis of *Filchneria*. Consequently, we transferred *S. zhouchangfai* into *Filchneria*. Moreover, we sequenced the COI sequences of four perlodids: *F. zhouchangfai*, *F. songi*, *Neowuia* sp. (undescribed species from Huo et al., in press) and *Stavsolus manchuricus* for building a phylogenetic tree based on three tribes, 17 genera and 30 species of subfamily Perlodinae. The position of *Filchneria* in tribe Perlodini, as well as the morphology and biogeography of *Filchneria* and related genera, are also discussed in this paper.

## 2. Materials and Methods

### 2.1. Sampling and Identification

The specimens were collected by hand and preserved in 75% ethanol. Abdominal segments of specimens were examined and illustrated using the KEYENCE VHX-5000 system. The materials are deposited in the Insect Collection of Yangzhou University (ICYZU), Jiangsu Province, China. The map of China in German is provided by Wikipedia (Available from: https://sk.wikipedia.org/wiki/Mal%C3%BD_Chingan, accessed on 12 March 2020); the modern map of China in this paper is modified by the National Platform for Common Geospatial Information Service (http://www.tianditu.gov.cn/, accessed on 13 March 2020 and 12 October 2022).

### 2.2. Preparation of Molecular Data

This study was conducted without harming protected or endangered species, and all research activities were authorized. The specimens of *Neowuia* sp. (in press), *Stavsolus manchuricus* and *Filchneria songi* were collected from Fujian, Liaoning and Shaanxi provinces, China, and preserved in 95% ethanol. Genomic DNA was extracted from the legs of specimens with the Column mtDNAout Kit (Axygen Biotechnology, Hangzhou, China) as recommended by the manufacturer and stored at −20 °C until used for PCR. PCR amplification and sequencing of the mitochondrial genome was amplified using LA-PCR and continuous specific PCR amplification according to the following conditions: performing initial denaturation at 93°C for 2 min and then performing 40 cycles at 92 °C for 10 s; annealing at 54 °C for 30 s; and stretching at 68 °C (20 cycles) at an 8 min elongation rate, which increases by 20 s/cycle in the last 20 cycles; the final extension is 10 min at 68 °C. PCR products were purified with Axygen DNA Gel Extraction Kit (Axygen Biotechnology, Hangzhou, China) [15], and quality control was subsequently carried out on the purified DNA samples. The quality of DNA was assessed using qubit3.0 and 1% agarose gel electrophoresis. High qualified DNA samples were applied to 500-bp paired-end library construction using the NEBNext Ultra DNA Library Prep Kit for Illumina sequencing. Sequencing was carried out on the Illumina NovaSeq 6000 platform (BIOZERON Co., Ltd., Shanghai, China). De novo assembly with GetOrganelle v1.6.4 referencing mitogenome of closely related species produced contigs of mitogenome. A number of potential mitochondrion reads were extracted from the pool of Illumina reads using BLAST searches against mitogenomes of related species and the GetOrganelle results. The mitochondrion Illumina reads were obtained to perform complete mitogenome de novo assembly using the SPAdes-3.13.1 package. The GetOrganelle assembly contig was optimized by the scaffolds from the SPAdes- 3.13.0 result. Finally, the assembled sequences were reordered and oriented according to the reference mitogenome, thus generating the final assembled mitochondrion genomic sequence (BIOZERON Co., Ltd., Shanghai, China) [16,17]. The entire COI genes are picked out of the PCG files with full length.

The phylogeny of COI genes of Perlodinae was analyzed, including *Filchneria zhouchangfai* (GenBank: ON023719), *Neowuia* sp. (GenBank: ON007241), *Stavsolus manchuricus* (GenBank: ON007243), *Filchneria songi* (GenBank: OM992360); *Arcynopteryx dichroa* (MZ607730.1), *Arcynopteryx compacta* (KU874207.1), *Skwala americana* (HQ568926.1), *Pseudomegarcys japonica* (LC644497.1), *Megarcys watertoni* (KM536888.1), *Megarcys subtruncata* (JF884135.1), *Setvena bradleyi* (JF884134.1), *Hemimelaena flaviventris* (MT407243.1), *Stavsolus spatulatus* (LC644498.1), *Yugus bulbosus* (HQ961195.1), *Yugus arinus* (HQ961194.1), *Malirekus hastatus* (HQ568925.1), *Isogenoides colubrinus* (HQ578977.1), *Isogenoides zionensis* (HQ961197.1), *Dictyogenus alpinus* (GU682169.1), *Dictyogenus fontium* (MZ027510.1), *Besdolus ravizzarum* (MF458629.1), *Besdolus imhoffi* (OK316455.1), *Besdolus ravizzarum* (MF458629.1), *Diura bicaudata* (MZ609905.1), *Diura nanseni* (MZ608587.1), *Perlodes dispar* (OK316486.1), *Perlodes microcephalus* (OK316440.1), *Hydroperla fugitans* (HQ971201.1) and *Hydroperla crosbyi* (HQ568946.1). *Amphinemura longispina* (NC044748.1) from another family was used as the outgroup species. All these data are downloaded from NCBI (https://www.ncbi.nlm.nih.gov/, accessed on 4 March 2022).

Sequence alignment and file format conversion was performed using MEGA 7.0.21. The best nucleotide substitution model was determined with MEGA 7.0.21 using the Bayesian Information Criterion (BIC) and the GTR+G+I model was predetermined for analyses. MrBayes v. 3.1.2 (http://morphbank.ebc.uu.SE/mrbayes/, accessed on 23 January 2022) was used with 1000 thousand generations to conduct Bayesian inference analysis; sampling was performed every 100 generations with four chains (three hot and one cold), and a burn-in of 25% trees. IQ-Tree v. 1.6.12 (http://www.iqtree.org/, accessed on 23 January 2022) was used for maximum likelihood with FigTree v. 1.4.2 (http://tree.bio.ed.ac.uk/software/figtree/, accessed on 23 January 2022) [18,19].

## 3. Morphological Taxonomy

### 3.1. Genus Filchneria Klapálek, 1908

*Skobeleva*: Klapálek, 1912, *Coll. Zool. Selys*. 4(1): 23.

*Filchneria*: Klapálek, 1908[1907], *Wiss. Zool. Botan. Ergebn*., 10: 61.

*Sinoperlodes*: Chen, 2020, *Zootaxa*, 4779 (4): 584–594. (nov. syn.)

### 3.2. Filchneria mongolica (Klapálek, 1901) (New Record in China)

*Dictyopteryx mongolica*: Klapálek, 1901, *Bull. Int. Acad. Sci. Bohême (Sci. Math. Nat.)*, 7:13.

*Filchneria mongolica*: Klapálek, 1908[1907] (partial misidentification), *Wiss. Zool. Botan. Ergebn*., 10: 61; Zwick, 1997, *Ephemeroptera & Plecoptera. Biology-Ecology-Systematics*, 489–496; Teslenko, Zwick and Bazova. 2010, *Zootaxa*, 2693: 50.

**Figure 3:**
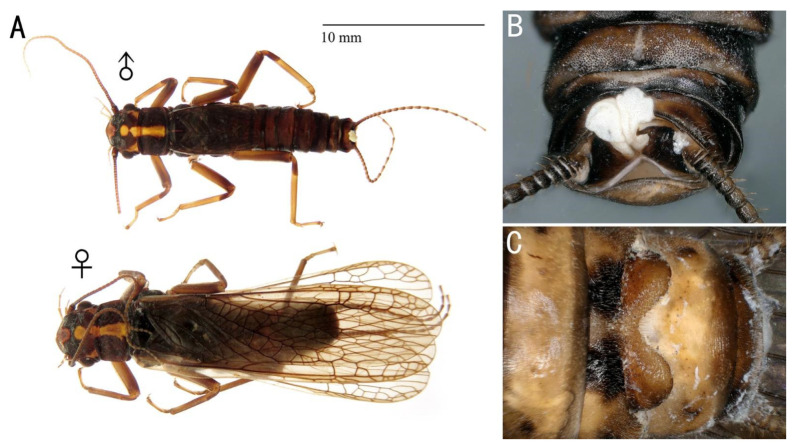
*Filchneria mongolica*. (**A**): male and female habitus; (**B**): male terminalia, dorsal; (**C**): female terminalia, ventral.

Description and data: See Teslenko et al. [13]. Adult habitus is shown in Figure 3.

Distribution: China (Arxan, Hinggan League); the transfrontier Selenga River in Mongolia and Russia (southern Siberia) and the Amur River Basin (Meun River, Bolshaya Ussurka River, Ussuri River) in the south of the Russian Far East.

Material examined: 2 ♂♂, 2 ♀♀, China, Arxan City, Hinggan League, Inner Mongolia Autonomous Region, 2018-V-28, leg. Xue Hai-Yang.

Remarks: The aedeagus of *F. mongolica* is short and flat; the paraproct is eversible; the basal sclerite is wide and well sclerotized. In comparison, *F. songi* has more complex aedeagal structures and is without eversible paraproct lobes.

This is not the first revision of this genus. The last revision was published by Teslenko et al. [13], who pointed out that the earlier redescription of *F. mongolica* by both Raušer [20] and Zhiltzova [21] is based on “the misidentified specimens”. While Zwick [22] and Teslenko et al. [13] redescribed the male *F. mongolica* and mentioned they are all brachypterous, but Klapálek [2] emphasized that “Beim ♂, welches voll entwickelte Flügel hat”, meaning the male from Qinling had fully developed wings (macropterous), fitting the feature of *F. songi*.

In all previous revisions, the Chinese specimens of Klapálek [2] were not available. According to the International Code of Zoological Nomenclature, the name *mongolica* has been used for over one century, and it seems impossible to be changed now, even though *F. songi* is the true type species. We have to accept the newest revision of Teslenko et al. [13] and maintain the current status of *F. mongolica* and *F. songi*. Otherwise, we would have to explain a more complex story about “how a type species was discovered in its type locality but published as a new species”.

### 3.3. Filchneria songi Chen, 2019

*Filchneria songi*: Chen, 2019, *Zootaxa*, 4623 (1): 189–200.

Description and data: see Chen [8].

**Figure 4:**
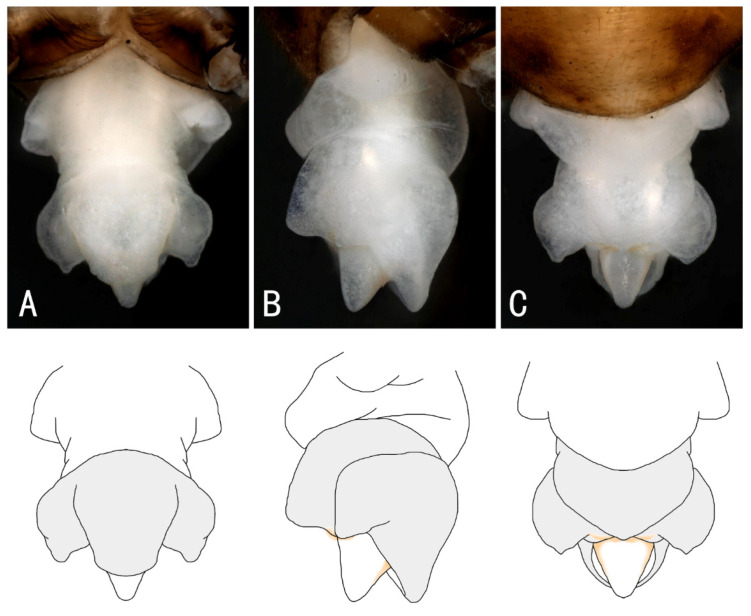
*Filchneria songi*, everted aedeagus. (**A**) dorsal; (**B**) lateral; (**C**) ventral.

Supplemental description: The aedeagus is a membranous, complex tridimensional structure and is translucent, without sclerite or spines, and smooth on the surface (Figure 4). The basal lobe and anterior lobe are inflated; the submedian is constricted (Figure 4A). The basal lobe has a pair of short tuberculous lateral arms (Figure 4A). The anterior lobe with a pair of large triangular lateral arms extends outwards; the dorsal part is swelling and hollowed ventrally as a hood-like cavity (Figure 4B). A short subulate process protrudes from the cavity, with a sparse brown hairbrush near the base (Figure 4C).

**Figure 5:**
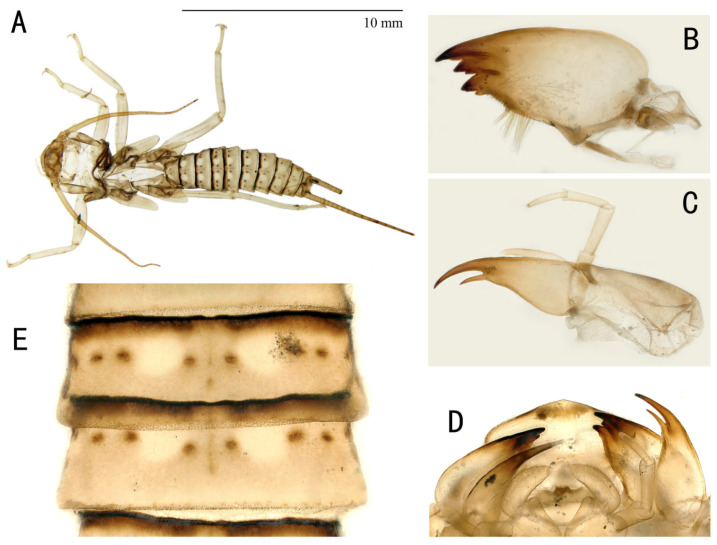
*Filchneria songi*, shed skin from Taiping National Forest Park. (**A**) nymph habitus; (**B**) mandible; (**C**) lacinia; (**D**) labium, ventral; (**E**) tergal stripes and dots.

Nymphs: Mature nymphs are 17–20 mm long (number = 5). Color of dorsal body is grey to olive green with some pale patterns (Figure 5A). Body is covered by short colorless hairs. The submental gill is almost absent. The mandible (Figure 5B) is bidentate and with three apical teeth which severally with three small subapical teeth; a patch of acanthae (14–18 hairs) is behind the last tooth. Lacinia (Figure 5C) is bidentate and apically narrow; the basal half is dramatically expanded; it is presented one to two setae below the base of the subapical tooth. There are two to three setae at the juncture of the apical teeth. The labium is shown in Figure 5. Thoracic segments are rectangular with broad, pale median stripes. Abdominal terga 1–8 have three pairs of large, distinct dark spots laterally and medially and two rows of large, oval, pale paramedial spots (Figure 5E). All abdominal segments have a few short, stout spinules on the posterior margin.

Material examined: 3 ♂♂, 5 ♀♀, 5 shed skin, the same locality and date as recorded in Chen [8].

**Figure 6:**
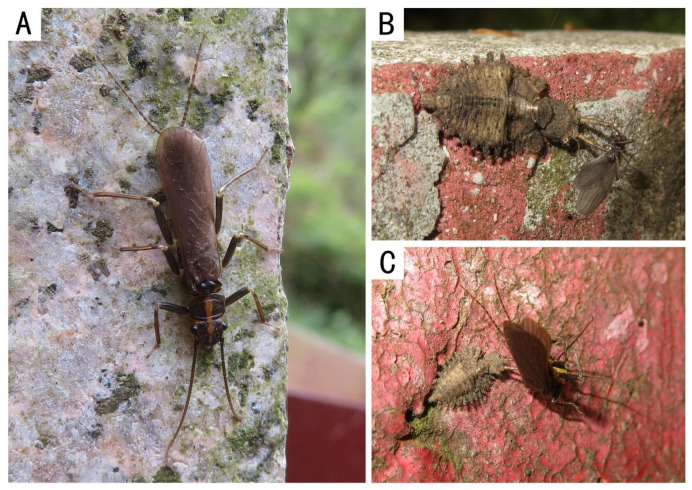
*Filchneria songi*. (**A**) male on the stone bridge; (**B**,**C**) the larvae of Ascalaphidae which were hunting Nemouridae sp. and *F. songi* on the nearby bridge.

**Figure 7:**
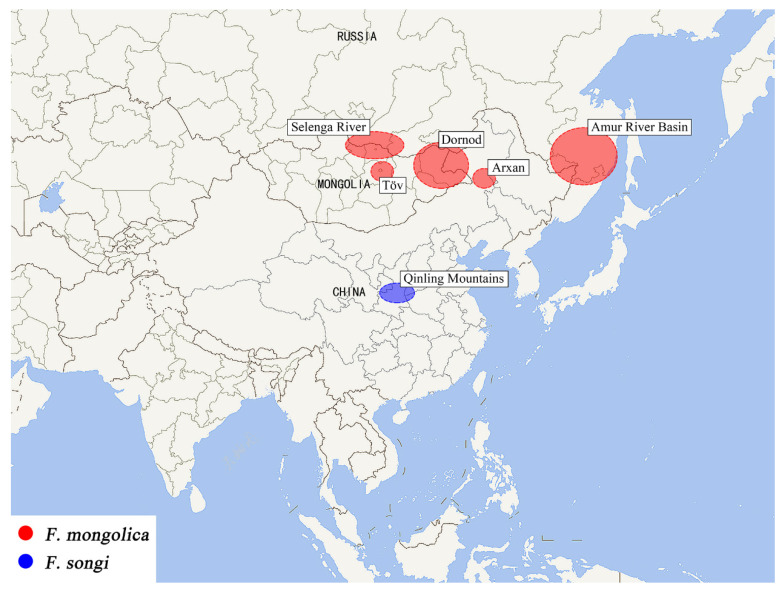
Distribution of *Filchneria mongolica* and *Filchneria songi* (map modified by www.tianditu.gov.cn, accessed on 12 October 2022).

Remarks: This species is only one of *Filchneria* known to emerge from March to April in the Qinling mountains. Adults are active during the day, crawling on the stone bridges near streams. The main predator of these stoneflies is the larva of Ascalaphidae, possibly *Maezous umbrosus* (Esben-Petersen, 1913), which also inhabits the same environment (Figure 6). The distribution range of *F. songi* and *F. mongolica* is shown in Figure 7.

### 3.4. Filchneria zhouchangfai (Chen, 2020) (comb. nov.)

*Sinoperlodes zhouchangfai*: Chen, 2020, *Zootaxa*, 4779 (4): 584–594.

**Figure 8:**
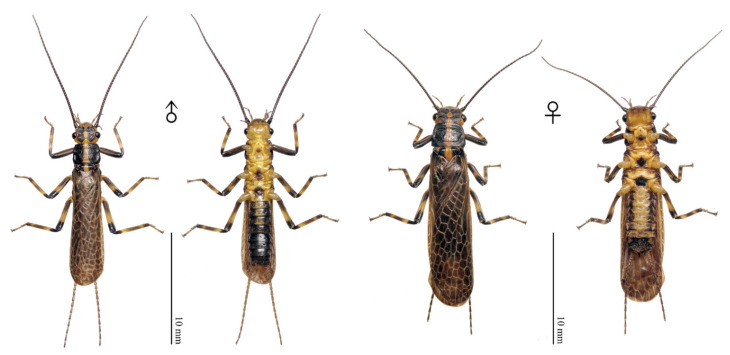
*Filchneria zhouchangfai* newly recorded from Anhui, male and female habitus.

Description and data: see Chen [14]. Adult habitus is shown in Figure 8.

**Figure 9:**
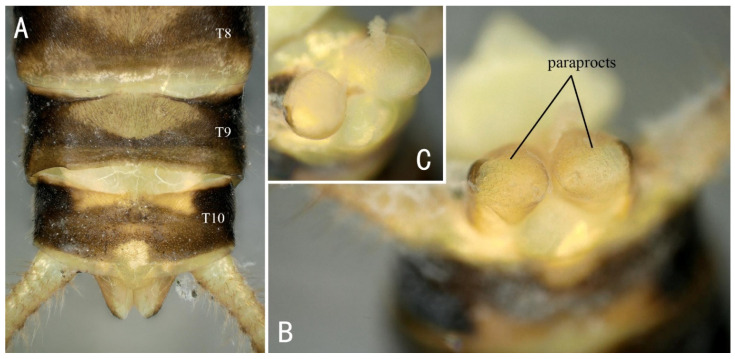
*Filchneria zhouchangfai* from Anhui, male. (**A**) terminalia, dorsal; (**B**) paraprocts; (**C**) paraprocts fully everted with liquid. T is for “tergum” (plural: terga).

**Figure 10:**
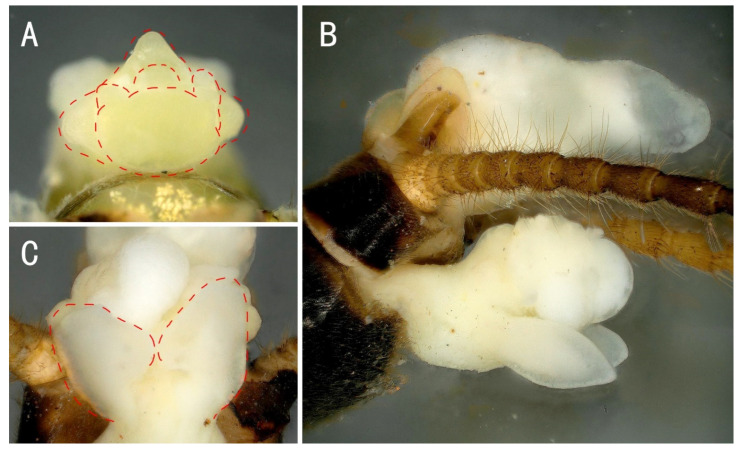
*Filchneria zhouchangfai* from Anhui, aedeagus. (**A**) top view; (**B**) lateral; (**C**) ventral.

Supplemental description: The front half of the paraproct is eversible and becomes swollen and hard when injected with bodily fluids (Figure 9). The aedeagus is a membranous, complex tridimensional structure; it is translucent, without sclerite or spines, and smooth on the surface. The basal lobe and anterior lobe are inflated; the submedian is constricted. The basal lobe has a pair of short tuberculous lateral arms. The anterior lobe has a pair of large triangular lateral arms extending outwards; the dorsal part is swelling and hollowed ventrally as a hood-like cavity. A short subulate process protrudes from the cavity, with a sparse brown hair brush near the base (Figure 10).

**Figure 11:**
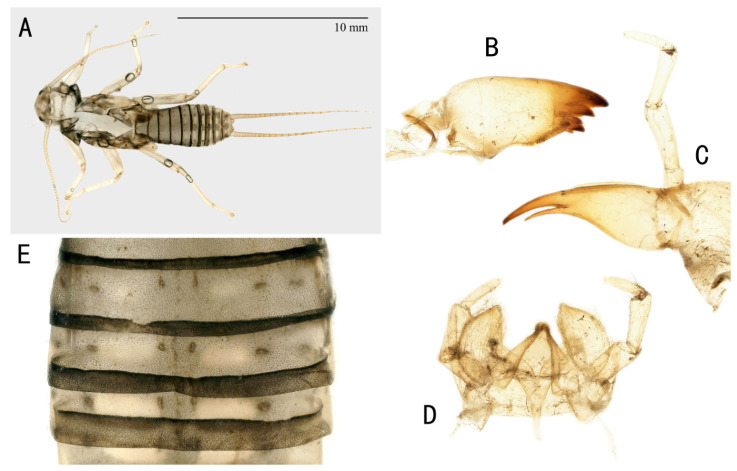
*Filchneria zhouchangfai* from Anhui, shed skin. (**A**) nymph habitus; (**B**) mandible; (**C**) lacinia; (**D**) labium, ventral; (**E**) tergal stripes and dots.

Nymphs: Mature nymphs are ca. 20 mm long (number = 9). Color of dorsal body is grey with some pale pattern; ventral body side is paler grey (Figure 11A). Body is covered by short colorless hairs. The mandible (Figure 11B) is bidentate with three apical teeth. The first and second teeth are without subapical teeth; the third teeth are combined with three small teeth (of which the median one is larger than the other two). The lacinia (Figure 11C) is bidentate and apically narrow; the basal half is dramatically expanded; it presents one to two setae below the base of the subapical tooth. There are two to three setae at the juncture of the apical teeth. The labium is shown in Figure 11D. Abdominal terga 1–8 have three pairs of small dark spots laterally and medially with two rows of oblate, pale paramedial spots. All abdominal segments have a few short, stout spinules on the posterior margin (Figure 11E). 

**Figure 12:**
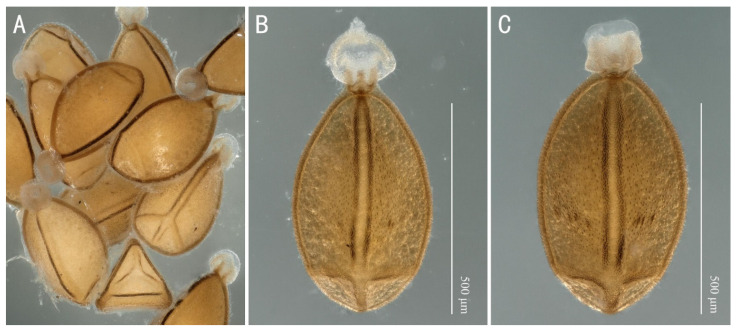
*Filchneria zhouchangfai* from Anhui, egg. (**A**) multiple eggs with different directions; (**B**,**C**) mature egg with few 1–2 or 4–5 micropyles.

Egg: The egg is trilateral. Longitudinal ridges delimit the three sides of the egg. Each side has additionally a transverse ridge close to the posterior pole (Figure 12A). The collar is long, formed by medially projecting extensions of the three longitudinal ridges, each flat and slender laterally and apex rounded (Figure 12B). A row of two to five micropyles is found near the transverse ridge (Figure 12B,C) on each of the three sides. The anchor is ball-shaped with a short pedicel with single globular bodies on the whole anchor plate. The margin of the anchor covers the collar completely. The structure of the chorion surface is rough with small light tubercles (Figure 12B,C).

Material examined: China: 2 ♂♂, 2 ♀♀, Zhejiang Province; ♂, Anhui Province, Huangshan City, Mt. Huangshan, “the Valley of Wild Monkeys”, 597 m, 30°5′11.508″ N, 118°8′29.7816″ E, 2020–IV–8, leg. Huo Qing-Bo, Zhu Bin-Qing; 7 ♂♂, 4 ♀♀, 9 shed skins, the same location in Mt. Huangshan, 2021–III–24~26, leg. Huo Qing-Bo, Zhao Meng-Yuan, Xiang Ya-Nan; ♂, Guizhou Province, Tongren City, Guanhe Township; 2020–IV, 2 ♂♂, 2021–III–8, the same location in Guanhe, leg. Hu Zheng-Kun; one adult with abdomen absent (dry specimen), Fujian Province, Wuyishan City, Mount Wuyi (Wuyishan), on the way from Saiyan to Tongmu Village, 726 m, 27°44′46″ N, 117°40′29″ E, 2021-VI-3, leg. Huo Qing-Bo, Zhu Bin-Qing.

Distribution: China (Zhejiang, Anhui, Fujian, Guizhou provinces).

Remarks: Morphologically, features of the male adult (terga and paraproct), egg (collar and transverse ridges), and nymph (mouthparts and abdominal pattern) of this species have no differences from *Filchneria*; especially the egg and nymph are more typically similar to *F. mongolica* and *F. songi*.

**Figure 13:**
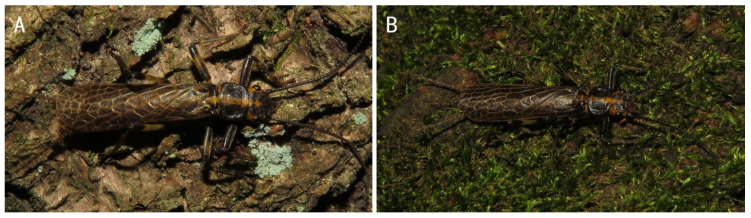
*Filchneria zhouchangfai* from Anhui, males on the tree trunks with few lichens (**A**) and multiple mosses (**B**).

In this study, all live samples of adults of this species were collected at night on tree trunks covered with moss or lichens (about 10 m from the river surface and about 4–5 m above the water surface), while the riverside stones and lights were never found on the lure. In addition, in Anhui and Zhejiang, we have seen two dying individuals that fell from the canopy (in which the male penis is partially exposed, which may have died naturally after mating). It suggests that these adults may be arboreal and do not have the same phototaxis as the local Perlidae species but may even be negative photokinetic like *Perlodinella* spp. (Huo et al. 2022, unpublished data) and *Capnia zijinshana* Du and Chen, 2016 [23]. The main diagnosis for this species being proposed as a new genus is the large area of transverse veins on the fore and hind wings of adults: in typical Perlodini, transverse veins are mainly concentrated on the leading edge of the wing but do not extend to the entire anal veins. Considering its special habit, we tend to think that its wing pattern are more like mimesis of the environment, or an evolution from the selective pressure by predators: the wings are interwoven with opaque black membranous areas and more transverse veins, which could reduce the reflection of light and make themselves look more mottled and inconspicuous when they are on the lichen and moss, whether the backgrounds are light (Figure 13A) or dark (Figure 13B). 

## 4. Phylogenetic Analysis

**Figure 14:**
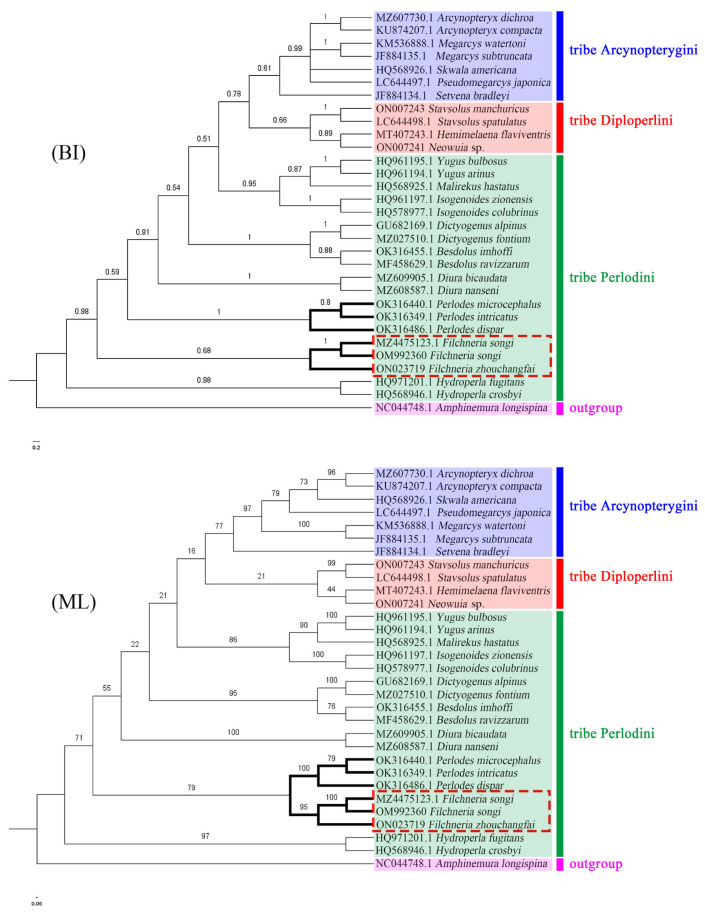
Phylogenetic relationships of Perlodinae, based on Maximum-Likelihood (ML) and Bayesian Inference (BI) methods. Numbers at the nodes represent posterior probabilities. Species names are marked to the right of each branch.

**Figure 15:**
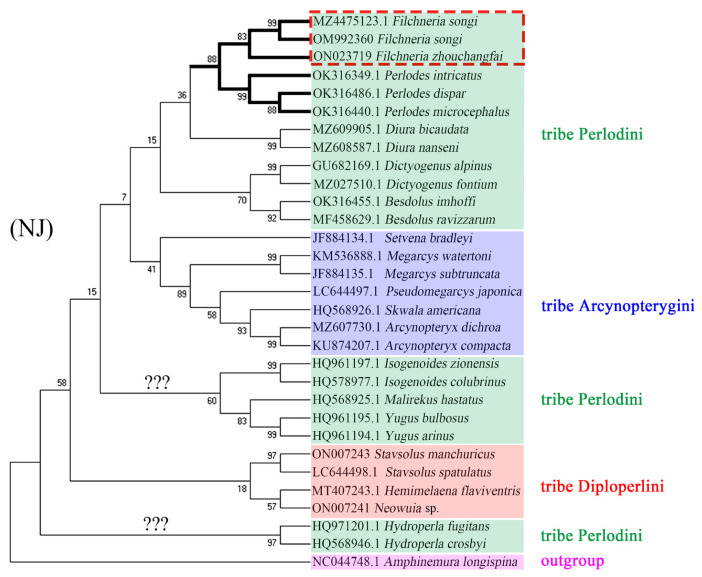
Phylogenetic relationships of Perlodinae, based on the neighbor-joining (NJ) method. The monophyly of the tribe Perlodini is not supported herein.

The phylogenetic analysis is based on the COI sequences of 17 genera and 30 species, and one Eugnathus (*Amphinemura longispina*) is listed as an outgroup. The results by Bayesian inference (BI), maximum likelihood (ML), and neighbor-joining (NJ) methods are roughly similar. BI and ML trees (Figure 14) match the current morphological classification of the subfamily Perlodinae. However, in the NJ tree (Figure 15), Diploperlini is included in the Perlodini, and the monophyly of Perlodini is not supported like the other two trees. Among all the results, only the support of the Diploperlini is generally low, probably because this tribe has the least data in this family, and more data will be needed to construct the phylogeny of Perlodinae in the future.

In the above results, there are two species of *Filchneria* (*F. zhouchangfai*, *F. songi*), and the three sequences are all clustered into one branch with high support and are most closely related to the genera *Perlodes* and *Hydroperla*. Both the *Filchneria* and *Perlodes* are shown as monophyletic, but only in the ML and NJ trees do the two genera come together to form a sister group. At present, there are few known data on *Filchneria* and *Perlodes*, and their phylogenetic relationship needs to be further studied. The above analysis results show that *F. zhouchangfai* is indeed a member of the *Filchneria* and should not be regarded as a separate genus, which is the same as the previous morphological identification results.

## 5. Discussion

### 5.1. Morphology

In the genera *Perlodinella*, *Perlodes*, and *Filchneria*, whether the epiproct of male can be everted, the morphology of male terminalia, and whether the basic segments of cerci have sclerotized teeth cannot be regarded as an absolute inter-generic diagnosis for the time being [22]. The morphological differences in the eggs of the above genera are sometimes even more obvious and more intuitive than the structure of their adults [13]. In addition, the descriptions of Perlodidae in the early literature are mostly very brief, and the epiproct or penis is often not well described. The morphology of most *Filchneria* species in China is still unclear and further research is needed.

**Figure 16:**
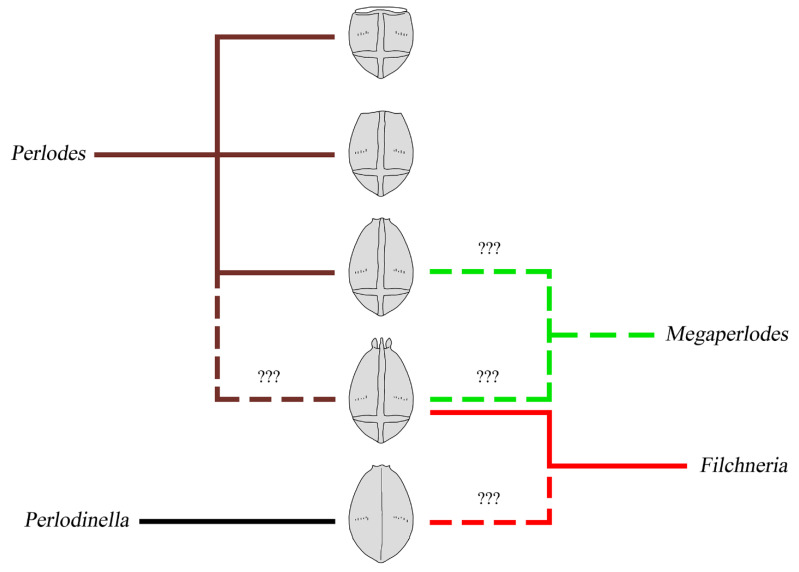
Egg morphology of four similar genera in Perlodini. The eggs of each genus are marked with a bold line in the recognized typical shapes and a dashed line in atypical shapes.

Teslenko et al. [13] pointed out that the genus *Filchneria* seems to be paraphyletic, and the species now placed in other genera may be *Filchneria*. For example, the highly curved tergum 10 and ridgeless eggs of *Filchneria wusuensis* Chen, 2019 and *Filchneria urumqiensis* Chen and Ma, 2021 [24,25] are totally consistent with the diagnosis of *Perlodinella*. Some other genera in Perlodini also face the same taxonomic confusion, such as the everted paraprocts and eggs of *Megaperlodes tiunovi* Teslenko, 2015 and *Perlodes stigmata* Ra, Kim, Kang and Ham, 1994, both of whose eggs have features typical of *Filchneria* [26]. Although the typical morphology of eggs of the European *Perlodes* is stable [27,28], there are also a small minority of species that are very similar to *Filchneria* and *Megaperlodes* [29] (Figure 16). Their few and fuzzy morphological differences suggest that the relationships of some genera in Perlodini need further study; *Filchneria*, *Perlodes* and *Perlodinella* seem to be all valid; only a few certain species need to be transferred from one into another, but *Megaperlodes* is perhaps a synonym of some genus above.

### 5.2. Biogeography

*Filchneria* undoubtedly shows a Palaearctic distribution and is widespread in the northern region of China. The subfamily Perlodinae is also only distributed in the Holarctic areas and has not been reported in the Oriental region, but two genera of Perlodinae (*Filchneria* and *Neowuia*) have been found in Fujian in recent years. In the research on insect biogeography, the boundary between the eastern part of the Palaearctic and the Oriental region has always been controversial: Ma [30] considered that the boundary of the two regions should be the Yangtze River (Jiangsu Province) or Hangzhou Bay (Zhejiang Province), but Shen [31] considered that the boundary could be further south, roughly in Fujian Province to northern Taiwan.

**Figure 17:**
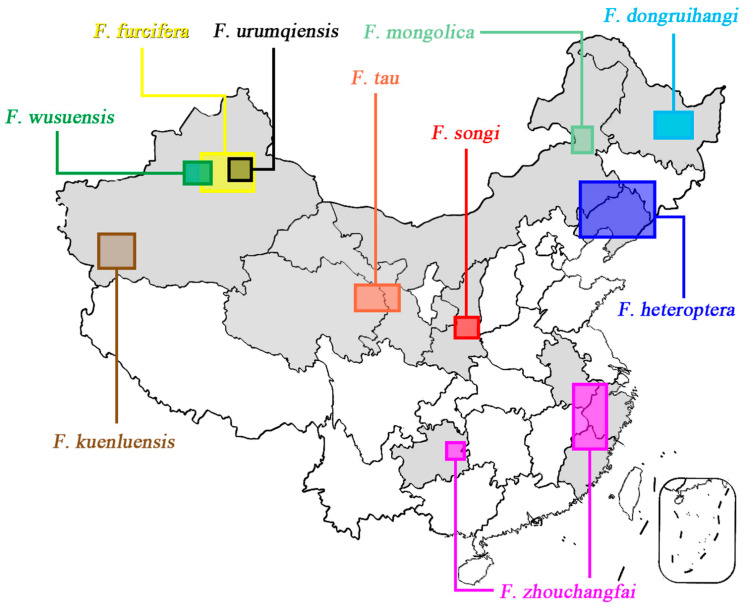
Distribution of *Filchneria* in China.

Huo [23] questioned the opinion in Shen [31] because his data about aquatic insects were indeed sparse for the calculation and analysis at that time, and the stonefly fauna in Jiangsu Province presents a large number of taxa that were initially considered to be typical of the Oriental distribution (genera *Flavoperla* Chu, 1929, *Kiotina* Klapálek, 1907, *Sinacroneuria* Yang and Yang, 1995, *Styloperla* Wu, 1935 et al.), and also includes other groups with a Palaearctic–Oriental distribution (*Togoperla* Klapálek, 1907, *Kamimuria* Klapálek, 1907) [4,23,32], so it is proposed to move the demarcation line northward from Hangzhou Bay to the southern Jiangsu (Nanjing to Yixing Cities) area. However, the opinion of Shen [31] seems to be further confirmed as more Perlodinae species are found in Zhejiang and Fujian than in the provinces with lower latitudes than Jiangsu (Figure 17). As far as the distribution of stoneflies is concerned, the Tianmu Mountains and the Wuyi Mountains may be regarded as transitional areas between the Palaearctic and Oriental regions.

**Figure 18:**
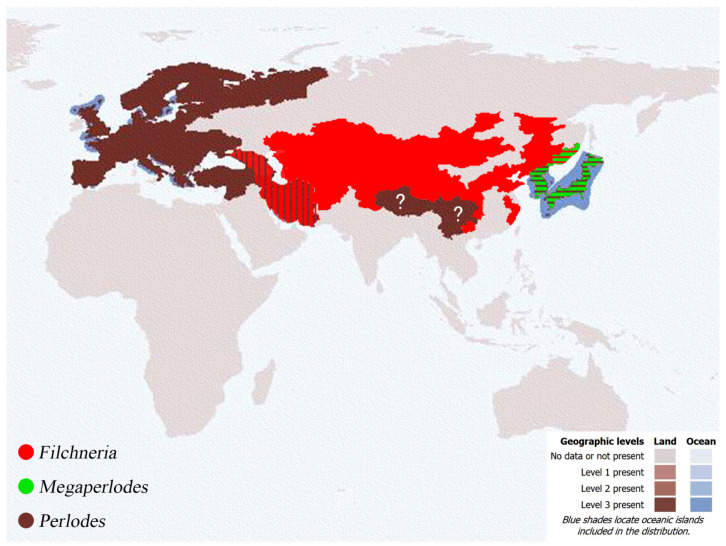
Distribution of *Filchneria, Megaperlodes* and *Perlodes*. Map modified according to Dewalt et al. [9], http://plecoptera.speciesfile.org, accessed on 12 October 2022.

The global distribution of the *Filchneria* and *Perlodes* appears to be naturally separated by the border between Asia and Europe, mainly the Ural Mountains and the Tibetan Plateau (Figure 18). *Filchneria* is widely distributed in East Asia, while *Perlodes* are mainly distributed in Europe and northern Asia, and they have a common distribution limited to the vicinity of Iran. In addition, the distribution record of *Perlodes* in western China [4] is still suspicious: all the Chinese *Perlodes* are dubious in taxonomy, because they are either poorly described or only female specimens (and not available) with obscure, poor descriptions [10]. Based on Figure 18, we tend to think that there is probably no distribution of *Perlodes* in China, and these species in Wu [4] should belong to other oriental genera of this subfamily.

## Data Availability

The molecular data presented in this study are downloaded from GenBank at https://www.ncbi.nlm.nih.gov/ (accessed on 12 October 2022), accession numbers ON023719, ON007241, ON007243 and OM992360.
